# Mycotoxins – Determination of deoxynivalenol and deepoxydeoxynivalenol in urine by LC-MS/MS: Biomonitoring Method – Translation of the German version from 2025

**DOI:** 10.34865/bi5148110e10_2or

**Published:** 2025-06-30

**Authors:** Marion Berger, Lennart Marske, Bernhard Monien, Solveigh Siodlaczek, Thomas Göen, Andrea Hartwig, MAK Commission

**Affiliations:** 1 Federal Institute for Occupational Safety and Health (BAuA). Division 4 – Hazardous Substances and Biological Agents. Unit 4.2 – Health Surveillance. Biological Monitoring Nöldnerstraße 40/42 10317 Berlin Germany; 2 German Federal Institute for Risk Assessment. Unit 54 – Food Safety Department Max-Dohrn-Straße 8–10 10589 Berlin Germany; 3 Friedrich-Alexander-Universität Erlangen-Nürnberg. Institute and Outpatient Clinic of Occupational, Social, and Environmental Medicine Henkestraße 9–11 91054 Erlangen Germany; 4 Institute of Applied Biosciences. Department of Food Chemistry and Toxicology. Karlsruhe Institute of Technology (KIT) Adenauerring 20a, Building 50.41 76131 Karlsruhe Germany; 5 Permanent Senate Commission for the Investigation of Health Hazards of Chemical Compounds in the Work Area. Deutsche Forschungsgemeinschaft, Kennedyallee 40, 53175 Bonn, Germany. Further information: Permanent Senate Commission for the Investigation of Health Hazards of Chemical Compounds in the Work Area | DFG

**Keywords:** Mykotoxine, Deoxynivalenol, Biomonitoring, Urin, LC-MS/MS, mycotoxins, deoxynivalenol, biomonitoring, urine, LC-MS/MS

## Abstract

The working group “Analyses in Biological Materials” of the German Senate Commission for the Investigation of Health Hazards of Chemical Compounds in the Work Area (MAK Commission) developed and verified the presented biomonitoring method. The aim of this method is the selective and sensitive quantitation of deoxynivalenol (DON; free DON plus glucuronides not otherwise specified) and its metabolite deepoxydeoxynivalenol (DOM‑1) in urine. After enzymatic hydrolysis of the urine sample and purification of the analytes on an immunoaffinity column, followed by preconcentration of the eluates under a stream of nitrogen, determination is carried out by high-performance liquid chromatography-tandem mass spectrometry (LC‑MS/MS). Calibration is performed with comparative standards prepared in urine and treated analogously to the samples to be analysed. DON is quantified using an internal standard (ISTD; ^13^C_15_‑DON), whereas DOM‑1 is quantified without the use of an ISTD. Good precision data with standard deviations below 9% for DON and below 6% for DOM‑1, as well as good accuracy data with mean relative recoveries in the range of 93–114% for DON and 97–103% for DOM‑1, show that the method provides reliable and accurate analytical results. The method is both selective and sensitive, and has a limit of quantitation of 0.179 μg/l for DON and of 0.26 μg/l for DOM‑1. Due to rapid renal excretion, the method is primarily suitable for analysing acute exposure which occurred only hours prior to sampling.

## 1 Characteristics of the method

**Table:** 

**Matrix**	Urine		
**Analytical principle**	Liquid chromatography with tandem mass spectrometry (LC‑MS/MS)		
**Parameters and corresponding hazardous substance**			
**Hazardous substance**	**CAS No.**	**Parameter**	**CAS No.**
Deoxynivalenol (DON)	51481-10-8	Deoxynivalenol (DON)	51481-10-8
		Deepoxydeoxynivalenol (DOM‑1)	88054-24-4

### Reliability criteria

#### Deoxynivalenol (DON)

**Table:** 

Within-day precision:	Standard deviation (rel.)	*s_w_* = 4.5%, 2.9%, 1.5%, 1.4%, or 1.1%
	Prognostic range	*u* = 11.5%, 7.5%, 3.9%, 3.7%, or 2.9%
	at a spiked concentration of 0.78 μg, 2.4 μg, 5.6 μg, 13.8 μg, or 16.8 μg DON per litre of urine and n = 6 determinations	
Day-to-day precision:	Standard deviation (rel.)	*s_w_* = 8.5%, 7.4%, 3.9%, 7.9%, or 2.1%
	Prognostic range	*u* = 20.1%, 17.4%, 10.0%, 19.2%, or 5.0%
	at a spiked concentration of 0.78 μg, 2.4 μg, 5.6 μg, 13.8 μg, or 16.8 μg DON per litre of urine and n = 6–8 determinations	
Within-day accuracy:	Recovery (rel.)	*r* = 103%, 111%, 98.8%, 114%, or 102%
	at a spiked concentration of 0.78 μg, 2.4 μg, 5.6 μg, 13.8 μg, or 16.8 μg DON per litre of urine and n = 6 determinations	
Day-to-day accuracy:	Recovery (rel.)	*r* = 93.3%, 102%, 94.9%, 104%, or 99.5%
	at a spiked concentration of 0.78 μg, 2.4 μg, 5.6 μg, 13.8 μg, or 16.8 μg DON per litre of urine and n = 6–8 determinations	
Limit of detection:	0.049 μg DON per litre of urine	
Limit of quantitation:	0.179 μg DON per litre of urine	

#### Deepoxydeoxynivalenol (DOM‑1)

**Table:** 

Within-day precision:	Standard deviation (rel.)	*s_w_* = 3.8%, 5.0%, or 4.6%
	Prognostic range	*u* = 9.8%, 12.9%, or 11.7%
	at a spiked concentration of 0.78 μg, 2.4 μg, or 5.6 μg DOM‑1 per litre of urine and n = 6 determinations	
Day-to-day precision:	Standard deviation (rel.)	*s_w_* = 3.2%, 5.1%, or 5.6%
	Prognostic range	*u* = 7.9%, 12.5%, or 14.3%
	at a spiked concentration of 0.78 μg, 2.4 μg, or 5.6 μg DOM‑1 per litre of urine and n = 6–7 determinations	
Within-day accuracy:	Recovery (rel.)	*r* = 102%, 101%, or 96.9%
	at a spiked concentration of 0.78 μg, 2.4 μg, or 5.6 μg DOM‑1 per litre of urine and n = 6 determinations	
Day-to-day accuracy:	Recovery (rel.)	*r* = 103%, 103%, or 96.6%
	at a spiked concentration of 0.78 μg, 2.4 μg, or 5.6 μg DOM‑1 per litre of urine and n = 6–7 determinations	
Limit of detection:	0.07 μg DOM‑1 per litre of urine	
Limit of quantitation:	0.26 μg DOM‑1 per litre of urine	

## 2 General information on deoxynivalenol

Mycotoxins are natural substances which are formed from fungi as secondary metabolic products. They are frequently present in edible fungus-infected plants (Eskola et al. 2020) and comprise a chemically and toxicologically heterogen­eous group of substances (Sabbioni et al. 2022), including aflatoxins, ochratoxin A, gliotoxin, citrinin (see also the method of the Commission: Berger et al. 2025) and deoxynivalenol (DON).

DON, also known as vomitoxin, is a mycotoxin of the trichothecene class and is formed from fungi, including the *Fusarium* genus, which affect sweet-grasses such as wheat, oats, barley, and corn (SCF 2002). DON inhibits protein synthesis by binding to ribosomes. Acute effects after ingestion of DON in humans include vomiting, diarrhoea, and dizziness. In animal studies in mice and pigs, growth retardation and dysregulation of the immune system were observed after chronic exposure (Pestka 2010).

**Fig.1:**
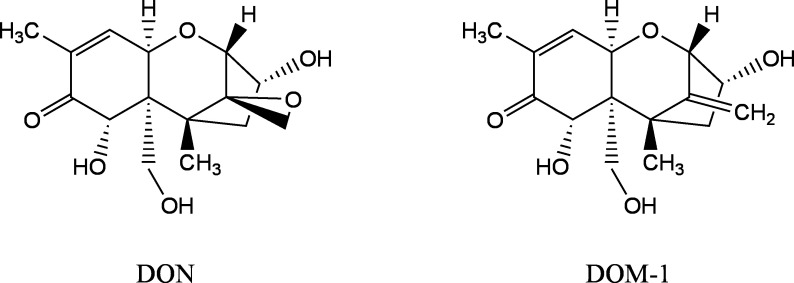
Structural formulas of DON and its metabolite DOM‑1

After oral ingestion, DON is completely absorbed in humans via the gastrointestinal tract and is completely renally excreted within 24 h, primarily in the form of the glucuronides DON‑15‑glucuronide (DON‑15‑GlcA) and DON‑3‑­glucuronide (DON‑3‑GlcA) (Mengelers et al. 2019; Warth et al. 2013). About 66–95% of DON is excreted as glucuronides, mainly as DON‑15‑GlcA (Vidal et al. 2018). Moreover, free DON and the metabolite deepoxydeoxynivalenol (DOM‑1) are excreted in the urine (Ali et al. 2016; Deng et al. 2018; Rodríguez-Carrasco et al. 2014). Figure 1 depicts the structural formulas of DON and DOM‑1.

Exposure in the general population via the consumption of DON‑contaminated food products has been frequently observed (Heyndrickx et al. 2015). The exposure of the European population to DON and its derivatives (3-acetyl-DON, 15-acetyl-DON and DON-3-glucoside) was analysed in the HBM4EU project, among others, due to the widespread occurrence and concerns about possible adverse effects on human health (Keyte et al. 2022; Namorado et al. 2024). The European Food Safety Authority (EFSA) has set a tolerable daily intake (TDI) of 1 μg/kg body weight for DON and its derivates (SCF 2002). Table 1 provides background concentrations of DON and DOM‑1 in urine from the general population.

**Tab.1:** Concentrations of DON and DOM‑1 in urine from the general population

Country/region (number of adults)	Sample	DON			DOM‑1			References
		Detection frequency [%]^a)^	GM/median [μg/l]	Range [μg/l]	Detection frequency [%]^a)^	Median [μg/l]	Range [μg/l]	
Belgium (239)	First morning void	37	1.7^c)^	< 0.5–130	–	–	–	Heyndrickx et al. 2015
Germany (120)	24‑h urine	98.3	2.66	0.69–17.05^b)^	–	–	–	Namorado et al. 2024
Germany (360)	24‑h urine	99	4.19	< 0.3–99.1	–	–	–	Schmied et al. 2023
Germany (50)	First morning void	100	7.35^c)^	1.06–38.4	–	–	–	Ali et al. 2016
Europe (1270)	Various urine samples	96.1	4.79	0.39–26.1^b)^	–	–	–	Namorado et al. 2024
Portugal (94)	24‑h urine	63	2.51^c)^	< 1.0–36.3	39	0.24	< 0.5–5.13	Martins et al. 2019
Spain (20)	First morning void	100	75.6^c)^	53.0–118.0^d)^	–	–	–	Gallardo-Ramos et al. 2024

GM: geometric mean

a) percentage of measured values above the quantitation limit

b) 5^th^–90^th^ percentile

c) Median

d) 25^th^–75^th^ percentile

In the workplace, exposure may occur via the inhalation of contaminated dusts and is described in agricultural production and food production (Föllmann et al. 2016; Turner et al. 2010; Viegas et al. 2018). Table 2 shows exemplary concentrations of DON and DOM‑1 in urine from occupationally exposed persons.

**Tab.2:** Concentrations of DON and DOM‑1 in urine from occupationally exposed persons

Exposed, country (n, sex)	Sample	DON			DOM‑1			References
		Detection frequency [%]^a)^	Mean ± SD [μg/l]	Range [μg/l]	Detection frequency [%]^a)^	Mean ± SD [μg/l]	Range [μg/l]	
Mill workers, Germany (12, ♂)	Spot urine	100	6.50 ± 3.33	3.28–13.8	54	0.105 ± 0.58^b)^	LOD (0.100)–0.216	Föllmann et al. 2016
Mill workers, Germany (5, ♀)	Spot urine	100	8.08 ± 4.48	0.850–10.4	60	0.110 ± 0.073^b)^	LOD (0.100)–0.228	
Controls, Germany (13, ♂)	Spot urine	100	6.85 ± 4.47	1.01–14.6	38	0.085 ± 0.049^b)^	LOD (0.100)–0.184	
Grain elevator workers, France (18)	First morning void	97	16.5^c)^	**–**	–	–	–	Ndaw et al. 2021
	Spot urine (pre-shift)	98	9.90^c)^	**–**	–	–	–	
	Spot urine (post-shift)	100	22.1^c)^	**–**	–	–	–	
Farmers, France (76, ♂)	First morning void	> 99	6.8^c)^	0.8−28.8	34	0.2^c)^	0.2–2.8	Turner et al. 2010
Workers in a fresh bread dough company, Portugal (9 ♀, 12 ♂)	Spot urine	43^d)^	34.9 ± 17.5^d), e)^	12.6−64.5^d), e)^	–	–	–	Viegas et al. 2018
Controls, Portugal (6 ♀, 12 ♂)	Spot urine	0	−	< LOD (1.24)–LOQ (4.14)^d), e)^	–	–	–	

LOD: limit of detection; LOQ: limit of quantitation; SD: standard deviation

a)  percentage of measured values above the LOQ

b)  Values < LOD were included as LOD/2 in the calculation of the mean value.

c)  median

d)  DON-glucuronide

e)  μg/g creatinine

## 3 General principles

The method described herein enables the quantification of the mycotoxin DON (free DON plus glucuronides not other­wise specified) and its metabolite DOM‑1 in urine. DON is quantified using an internal standard (ISTD; ^13^C_15_‑DON), whereas DOM‑1 is quantified without the use of an ISTD. After enzymatic hydrolysis of the urine sample, the analytes are enriched on an immunoaffinity column and eluted with methanol. The eluates are then concentrated under a stream of nitrogen and the analytes are subsequently measured by high-performance liquid chromatography-tandem mass spectrometry (LC‑MS/MS). Calibration is performed with comparative standards prepared in urine and treated analogously to the samples to be analysed.

## 4 Equipment, chemicals, and solutions

### 4.1 Equipment

- HPLC system with a binary pump, autosampler, column oven, and degasser (e.g. Nexera XR, Shimadzu Deutschland GmbH, Duisburg, Germany)
- Triple-quadrupole mass spectrometer (e.g. AB SCIEX QTRAP 5500 with electrospray ionisation, AB SCIEX Germany GmbH, Darmstadt, Germany)
- Analytical HPLC separation column (e.g. Kinetex^®^ Core-Shell technology; Kinetex^®^ 2.6 μm biphenyl 100 Å, 100 × 2.1 mm, Phenomenex Ltd. Deutschland, Aschaffenburg, Germany)
- UHPLC precolumn (e.g. No. AJO-9209, SecurityGuard ULTRA Cartridges, biphenyl 2.1 mm ID, including column holder, Phenomenex Ltd. Deutschland, Aschaffenburg, Germany)
- Nitrogen generator (e.g. cmc Instruments GmbH, Eschborn, Germany)
- Water-purification system (e.g. Veolia Water Solutions & Technologies, Saint-Maurice, France)
- Laboratory centrifuge (e.g. Fisher Scientific GmbH, Schwerte, Germany)
- Analytical balance (e.g. Sartorius AG, Göttingen, Germany)
- pH meter (e.g. Mettler-Toledo GmbH, Gießen, Germany)
- Evaporator (e.g. Biotage Sweden AB, Uppsala, Sweden)
- Ultrasonic bath for degassing the eluents (e.g. SONOREX SUPER RK 510 H, BANDELIN electronic GmbH & Co. KG, Berlin, Germany)
- Tube Rotator (e.g. Cole-Parmer^TM^ Stuart^TM^, Fisher Scientific GmbH, Schwerte, Germany)
- Vortex shaker (e.g. IKA‑Werke GmbH & Co. KG, Staufen, Germany)
- Vacuum unit (e.g. VisiPrep^TM^ SPE Vacuum Manifold, Supelco^®^, Merck KGaA, Darmstadt, Germany)
- Incubator with orbital shaker (e.g. Cole-Parmer^TM^ Stuart^TM^, Fisher Scientific GmbH, Schwerte, Germany)
- Variably adjustable pipettes with matching pipette tips (e.g. Eppendorf AG, Hamburg, Germany)
- 2500‑ml glass bottles with screw cap (e.g. DURAN^®^, Schott AG, Mainz, Germany)
- Various volumetric flasks and glass beakers (e.g. DURAN^®^, Schott AG, Mainz, Germany)
- 0.2‑μm syringe filter (13 mm, regenerated cellulose) (e.g. CS – Chromatographie Service GmbH, Langerwehe, Germany)
- 15‑ml polypropylene conical centrifuge tubes, graduated (e.g. COTECH Vertriebs GmbH, Berlin, Germany)
- 13‑ml polypropylene round-bottom centrifuge tubes (e.g. COTECH Vertriebs GmbH, Berlin, Germany)
- 5‑ml disposable Luer-Lock syringes with disposable injection cannulae (e.g. Omnifix^®^ Luer Solo, B. Braun SE, Melsungen, Germany)
- 1.5‑ml polypropylene threaded vials with screw caps (e.g. MACHEREY-NAGEL GmbH & Co. KG, Düren, Germany)
- 2‑ml polypropylene microcentrifuge tubes (e.g. Eppendorf AG, Hamburg, Germany)
- Urine cups made of polypropylene (e.g. Sarstedt AG & Co. KG, Nümbrecht, Germany)

### 4.2 Chemicals

Unless otherwise specified, all chemicals must be a minimum of *pro analysi* grade.

#### Reference standards and ISTD

- Deoxynivalenol (DON), 100 mg/l in acetonitrile (e.g. No. 34124, Supelco^®^, Merck KGaA, Darmstadt, Germany)
- Deepoxydeoxynivalenol (DOM‑1), 50 mg/l in acetonitrile (e.g. No. 10003662 (S02033), Romer Labs Division Holding GmbH, Getzersdorf, Austria)
- ^13^C_15_‑Deoxynivalenol, 25 mg/l in acetonitrile (e.g. No. DRE-A12147100AL-25, LGC Standards GmbH, Wesel, Germany)

#### Other chemicals

- Immunoaffinity column IAC DONStarR, storage at 4 °C (e.g. No. 10001970, Romer Labs Division Holding GmbH, Getzersdorf, Austria)
- Ammonium acetate (e.g. No. 15681570, Honeywell Fluka^TM^, Fisher Scientific GmbH, Schwerte, Germany)
- Disodium hydrogen phosphate dihydrate (e.g. No. 137036, Merck KGaA, Darmstadt, Germany)
- *Escherichia coliβ*‑glucuronidase, K12, ≥ 140 U/mg at 37 °C (e.g. No. 03708446103, Roche Diagnostics Deutschland GmbH, Mannheim, Germany)
- Acetic acid LiChropur^®^, 100% (e.g. No. 533001, Supelco^®^, Merck KGaA, Darmstadt, Germany)
- Isopropanol LiChrosolv^®^, to clean the posterior pistons of the pumps, among other uses (e.g. No. 102781, Supelco^®^, Merck KGaA, Darmstadt, Germany)
- Potassium dihydrogen phosphate (e.g. No. 137039, Merck KGaA, Darmstadt, Germany)
- Methanol LiChrosolv^®^, ≥ 99.97% (e.g. No. 106035, Supelco^®^, Merck KGaA, Darmstadt, Germany)
- PBS tablets Calbiochem^®^ (e.g. No. 524650, Merck KGaA, Darmstadt, Germany)
- Ultra-pure water (e.g. Veolia Water Solutions & Technologies, Saint-Maurice, France)
- Native urine from volunteers with DON and DOM‑1 levels which are as low as possible

### 4.3 Solutions

- Stock solution A for phosphate buffer according to Sørensen (pH 6.8)
  9.078 g potassium dihydrogen phosphate are weighed into a 1000‑ml volumetric flask and dissolved in a little ultra-pure water. Subsequently, the flask is made up to the mark with ultra-pure water and the solution is thoroughly mixed.
- Stock solution B for phosphate buffer according to Sørensen (pH 6.8)
  11.876 g disodium hydrogen phosphate dihydrate are weighed into a 1000‑ml volumetric flask and dissolved in a little ultra-pure water. Subsequently, the flask is made up to the mark with ultra-pure water and the solution is thoroughly mixed.
- Phosphate buffer according to Sørensen (pH 6.8)
  49.2 ml of stock solution B are pipetted into a 100‑ml volumetric flask. Subsequently, the flask is made up to 100 ml with stock solution A and the solution is thoroughly mixed.

The phosphate buffer according to Sørensen is stable at 4 °C for at least one week.

- Eluent A
  77.08 mg ammonium acetate are weighed into a 1000‑ml volumetric flask and dissolved in a little ultra-pure water. Subsequently, 1 ml of acetic acid is pipetted into the flask, which is then made up to the mark with ultra-pure water.
- Eluent B
  77.08 mg ammonium acetate are weighed into a 1000‑ml volumetric flask and dissolved in a little methanol. Subsequently, 1 ml of acetic acid is pipetted into the flask, which is then made up to the mark with methanol.
- Gradient solution (Eluent A ∶ Eluent B; 98 ∶ 2 (v ∶ v))
  2 ml of Eluent B are placed in a 100‑ml volumetric flask, which is then made up to the mark with Eluent A.
- PBS buffer (pH 7.4)
  One PBS tablet is placed in an 800‑ml glass beaker and about 500 ml of ultra-pure water are added. The glass beaker is then set in an ultrasonic bath until the tablet has completely dissolved. Subsequently, the solution is transferred into a 1000‑ml volumetric flask, rinsing the beaker several times with ultra-pure water. Finally, the volumetric flask is made up to the mark with ultra-pure water and the solution is thoroughly mixed.

The PBS buffer is stable at room temperature for at least six months.

### 4.4 ISTD

- ^13^C_15_‑DON spiking solution (150 μg/l)
  In a 2‑ml polypropylene microcentrifuge tube, 6 μl of the reference-standard solution for ^13^C_15_‑DON (25 μg/ml in acetonitrile) are mixed with 994 μl of ultra-pure water and the solution is thoroughly mixed.

The ^13^C_15_‑DON spiking solution is stable for four days when stored at −20 °C.

### 4.5 Calibration standards

- Calibration-standard spiking solution (150 μg analyte/l)
  1.5 μl of the DON standard solution (100 mg/l) and 3 μl of the DOM‑1 standard solution (50 mg/l) are pipetted into a 2‑ml polypropylene microcentrifuge tube and mixed with 995 μl of ultra-pure water, then the solution is thoroughly mixed.

The calibration-standard spiking solution is stable for four days when stored at −20 °C.

The calibration standards are prepared according to the pipetting scheme given in Table 3. The calibration-standard spiking solution is added to 2.5 ml of preferably unexposed native urine in a 13‑ml propylene round-bottom centrifuge tube. The urine used to prepare the calibration standards should have the lowest possible background levels of DON and DOM‑1. The developers of the method used the urine of a volunteer who had abstained from eating cereal grain products for five days.

**Tab.3:** Pipetting scheme for the preparation of calibration standards for the determination of DON and DOM‑1 in urine

Solution	Calibration-standard spiking solution [μl]	Analyte concentration [μg/l]	^13^C_15_‑DON spiking solution [μl]	^13^C_15_‑DON concentration [μg/l]
DB^a)^	0	0	0	0
B^b)^	0	0	25	1.5
C1	5	0.3	25	1.5
C2	10	0.6	25	1.5
C3	15	0.9	25	1.5
C4	20	1.2	25	1.5
C5	25	1.5	25	1.5
C6	35	2.1	25	1.5
C7	50	3	25	1.5
C8	100	6	25	1.5
C9	150	9	25	1.5
C10	200	12	25	1.5
C11	250	15	25	1.5
C12	300	18	25	1.5
C13	350	21	25	1.5

a) double-blank

b) blank

### 4.6 Control-standard solution

To test the equilibrated measuring system, measurement of a control‑standard solution is used (testing for pressure, peak intensity and retention times). Control‑standard solutions are analysed before and after the samples of a series.

- Control‑standard solution (7 μg DON/DOM‑1/^13^C_15_‑DON/l)
  In a 1.5‑ml polypropylene threaded vial with screw cap, 46.7 μl of the calibration‑standard spiking solution (150 μg/l) and 46.7 μl of the ^13^C_15_‑DON spiking solution (150 μg/l) are added to 907 μl of the gradient solution, then the solution is thoroughly mixed.

The control‑standard solution is stored at −20 °C and freshly prepared every week.

## 5 Specimen collection, sample preparation, and purification with the immunoaffinity column

### 5.1 Specimen collection

The urine samples are collected in polypropylene urine cups, aliquoted, and stored at −20 °C until sample preparation.

### 5.2 Sample preparation

Prior to sample preparation, the urine samples are brought to room temperature and homogenised. 2.5 ml of the urine samples are placed in 13‑ml round-bottom centrifuge tubes, mixed with 25 μl of the ^13^C_15_‑DON spiking solution, and thoroughly mixed. For enzymatic hydrolysis, samples are then mixed with 2.5 ml of the phosphate buffer (pH 6.8) and 40 μl of *β*‑glucuronidase, then shaken for 20 h in an incubator at 37 °C and 210 rotations/min. After hydrolysis, the samples are centrifuged at 2045 × *g* and 10 °C for 15 min. The supernatants are decanted into new 13‑ml round-bottom centrifuge tubes.

### 5.3 Purification with the immunoaffinity column

The preconcentration of DON and DOM‑1 is carried out on an immunoaffinity column combined with an SPE vacuum-chamber system. The stationary phase of the immunoaffinity column is a gel to which analyte-specific antibodies are coupled. It is not necessary to condition the column before loading the urine sample. After bringing the column and the urine sample to room temperature, the sample is applied in portions onto the column for enrichment and purification. Sample application is carried out without the use of a vacuum. The flow rate is about 1 ml/min. The column is subsequently washed with 2 × 2.5 ml of PBS buffer. After washing, any liquid residue remaining in the column is removed under light pressure from above, whereby the column may not be allowed to dry out. Anhydrous methanol is used for the elution of the bound analytes. Elution is performed with 2 × 1.5 ml methanol into a graduated 15‑ml conical centrifuge tube into which 200 μl of ultra-pure water have been placed. The first 1.5 ml of methanol are left on the column for a few seconds before elution. Any methanol residues remaining in the column are eluted by applying a slight overpressure. At 40 °C, the sample is evaporated to 200 μl under a stream of nitrogen, mixed with 300 μl of gradient solution, homogenised with a vortex shaker, and then filtered into a 1.5‑ml polypropylene threaded vial with screw cap via a 0.2‑μm syringe filter.

## 6 Operational parameters

Analytical determination was carried out using a device configuration comprised of a liquid chromatograph (Nexera XR, Shimadzu Deutschland GmbH, Duisburg, Germany) and a tandem mass spectrometer (AB SCIEX QTRAP 5500, AB SCIEX Germany GmbH, Darmstadt, Germany).

### 6.1 High-performance liquid chromatography

**Table:** 

HPLC column:	Kinetex^®^ biphenyl; 2.6 μm; 100 × 2.1 mm
Precolumn:	UHPLC precolumn biphenyl 2.1 mm ID
Column-oven temperature:	40 °C
Autosampler temperature:	15 °C
Injection volume:	20 μl
Eluent A:	0.1% acetic acid and 1 mM ammonium acetate in ultra-pure water
Eluent B:	0.1% acetic acid and 1 mM ammonium acetate in methanol
Gradient programme:	see Table 4

**Tab.4:** Gradient programme for the determination of DON and DOM‑1 in urine

Time [min]	Flow rate [ml/min]	Eluent A [%]	Eluent B [%]
0.01	0.45	98	2
2	0.45	98	2
5	0.45	20	80
5.2	0.45	2	98
8	0.45	2	98
8.01	0.45	98	2
11	0.45	98	2

### 6.2 Tandem mass spectrometry

**Table:** 

Source:	TurboSpray
Ionisation mode:	ESI, negative
Ion-spray voltage:	−4500 V
Source temperature:	500 °C
Nebulising gas:	Nitrogen, 80 psi (551.58 kPa)
Turbo-heater gas:	Nitrogen, 80 psi (551.58 kPa)
Curtain gas:	Nitrogen, 35 psi (241.32 kPa)
Collision gas:	Nitrogen
Scan mode:	*Multiple Reaction Monitoring* (MRM)
Dwell time:	70 msec
Parameter-specific settings:	see Table 5

The retention times given in Table 5 can only serve as a point of reference. The user must ensure the separation performance of the column used and the resulting retention behaviour of the analytes.

**Tab.5:** MRM parameters and retention times for the determination of DON and DOM‑1 in urine

Analyte/ ISTD	Retention time [min]	Precursor ion (***m/z***)	Product ion (***m/z***)	Declustering potential [V]	Entrance potential [V]	Collision energy [V]	Cell-exit potential [V]
DON	4.81	355.2	59^a)^	−45	−10	−52	−5
			295.1	−45	−10	−14	−19
			265.1	−45	−10	−20	−17
DOM‑1	5.12	339.2	59^a)^	−70	−10	−20	−9
			249.2	−70	−10	−16	−17
^13^C_15_‑DON	4.81	370.3	59.1^a)^	−40	−10	−40	−7
			310.3	−40	−10	−14	−29

a) Quantifier

## 7 Analytical determination

For the analytical determination of DON and DOM‑1 in urine, 20 µl of each of the samples prepared according to Section 5 are injected into the HPLC‑MS/MS system and analysed under the conditions specified in Section 6. The analytes are separated after passing through a UHPLC biphenyl precolumn on a Kinetex^®^ biphenyl column. The analytes DON and DOM‑1 are identified by their retention times and characteristic mass transitions (see Table 5).

**Fig.2:**
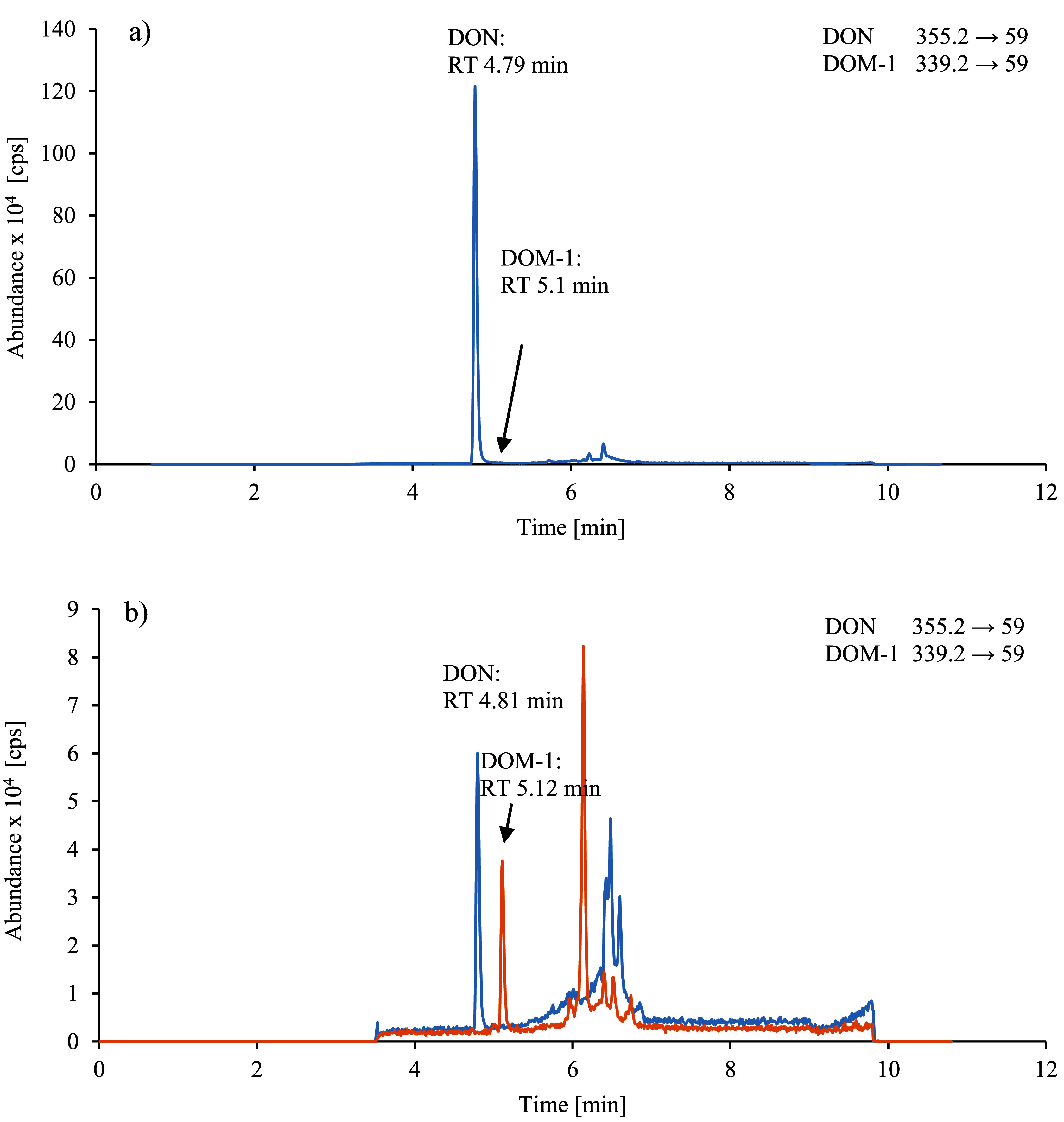
Chromatogram a) of a native urine sample with 14.4 μg DON/l and a DOM‑1 concentration below the quantitation limit; and b) of a calibration standard with 0.3 μg DON and 0.3 µg DOM‑1 per litre of urine

Figure 2 depicts representative chromatograms of a) a native urine sample with a determined DON concentration of 14.4 μg/l urine as well as a DOM‑1 concentration below the quantitation limit and b) a calibration standard for DON and DOM‑1 with concentrations of 0.3 μg/l urine, each.

## 8 Calibration

For the calibration of the method, the calibration solutions prepared according to Section 4.5 are processed analogously to the urine samples (cf. Section 5), although without further addition of ISTD, and analysed by HPLC‑MS/MS (cf. Section 6). The calibration curve for DON is generated by plotting the peak-area ratio of DON and ^13^C_15_‑DON against the concentration ratio of DON and ^13^C_15_‑DON. The calibration curve for DOM‑1 is generated by plotting the peak area against the spiked concentration of DOM‑1.

**Fig.3:**
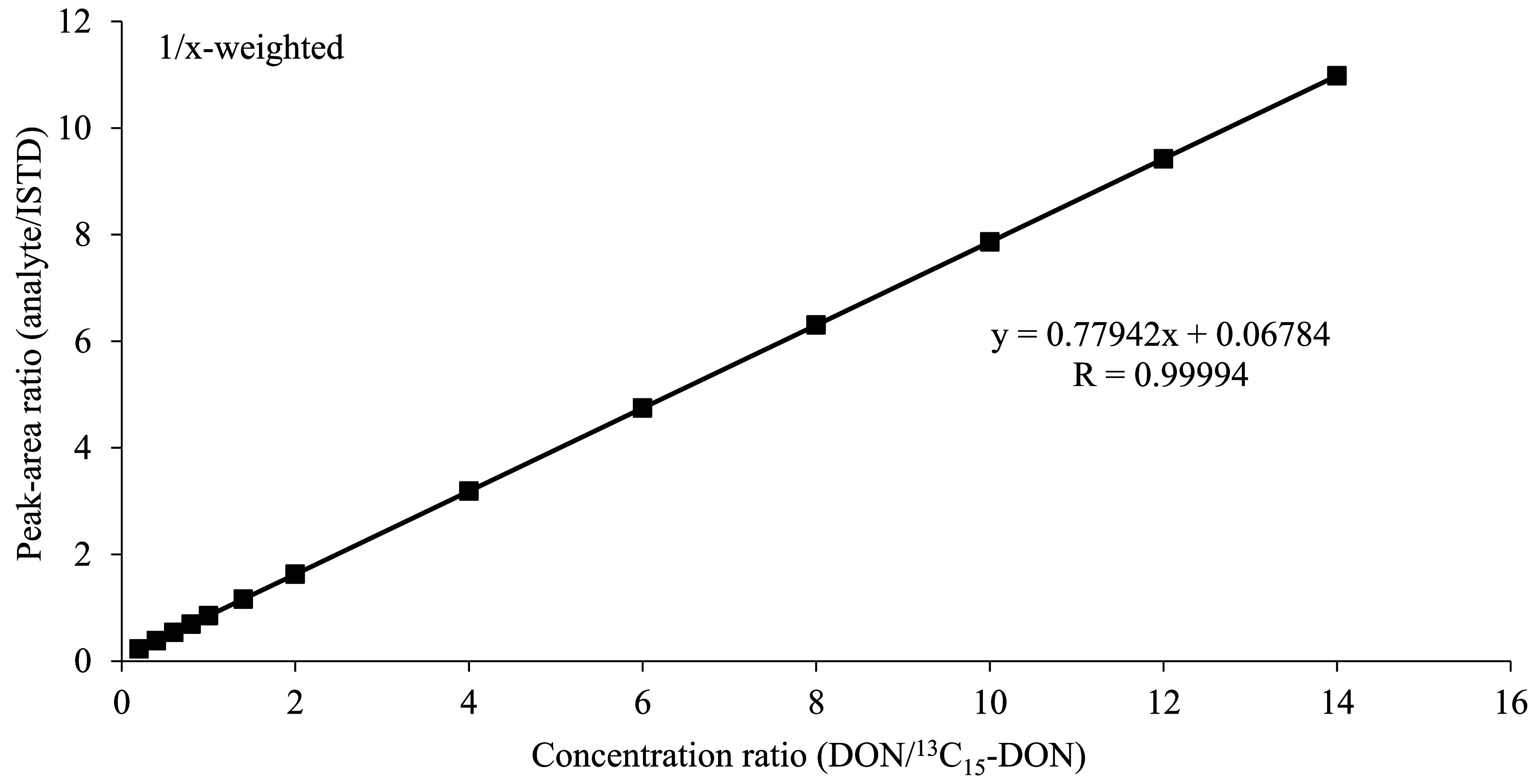
Calibration curve for the determination of DON in urine

**Fig.4:**
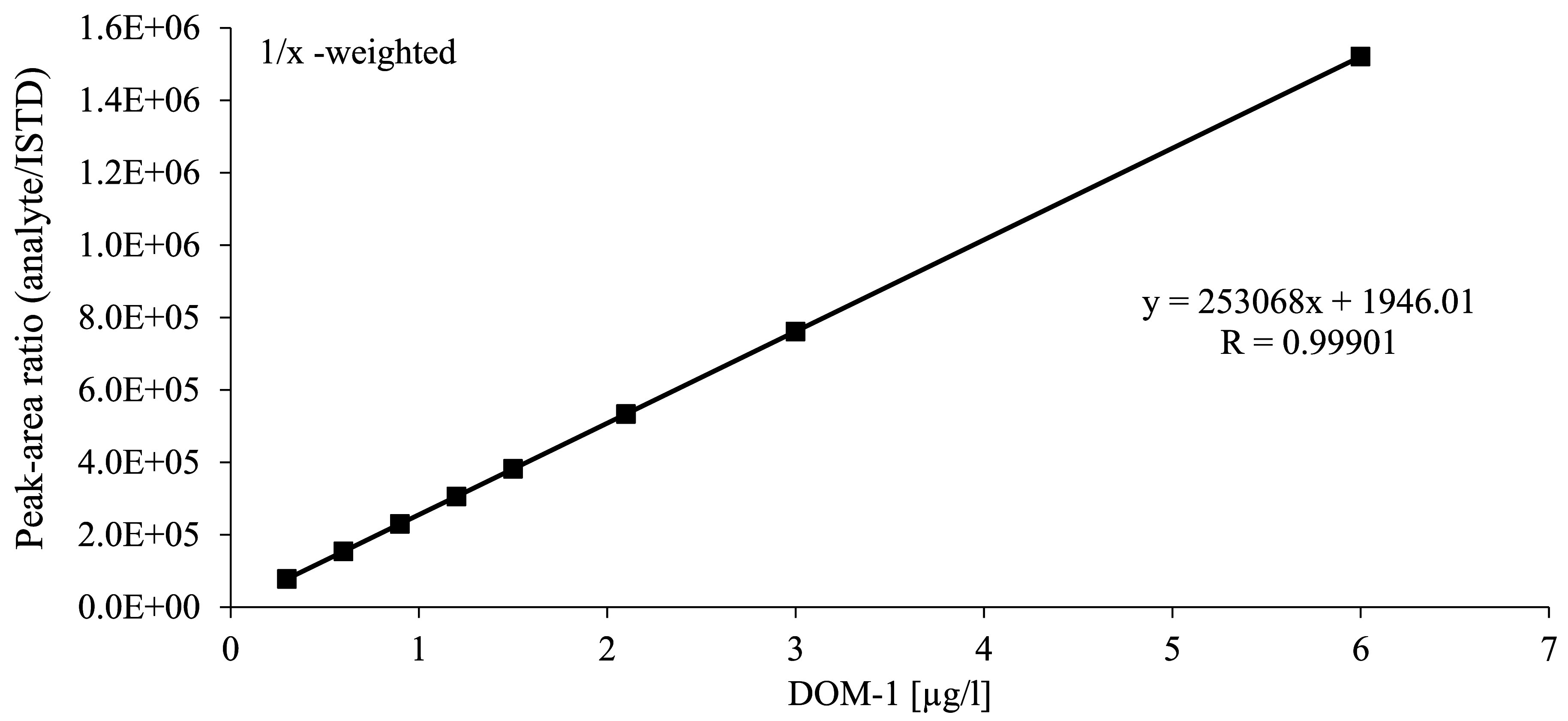
Calibration curve for the determination of DOM‑1 in urine

The individual calibration ranges are given in Table 6. The data were fitted using a linear function with 1/x-weighting. For both analytes, correlation coefficients of R ≥ 0.999 were found in the concentration ranges investigated. Figure 3 and 4 depict representative calibration curves for the determination of DON and DOM‑1 in urine.

**Tab.6:** Calibration ranges for the determination of DON and DOM 1 in urine

Analyte	Calibration range [μg/l]	ISTD	ISTD [μg/l]
DON	0.3–21	^13^C_15_‑DON	1.5
DOM‑1	0.3–6	–	–

## 9 Calculation of analytical results

To calculate the concentration of DON in a urine sample, the peak area of the analyte is divided by the peak area of the ISTD ^13^C_15_‑DON. With the calibration function corresponding to the analytical run in question (cf. Section 8), the quotient thus obtained can be used to calculate the analyte concentration in μg/l urine.

To calculate the concentration of DOM‑1 in a urine sample, the determined peak area and the calibration function corresponding to the analytical run in question (cf. Section 8) are used to calculate the analyte concentration in μg/l urine.

The calibration range may need to be adjusted to the expected concentration ranges.

## 10 Standardisation and quality control

Quality assurance of the analytical results is carried out as stipulated in the guidelines of the *Bundesärzte­kammer* (German Medical Association) and in a general chapter published by the Commission (Bader et al. 2010; Bundes­ärztekammer 2014).

For quality control, blank and double-blank samples are prepared in urine with every calibration series (see Table 3). The blank samples are spiked with ISTD but not with the analytes, the double-blank samples contain neither analyte nor ISTD. In addition, a reagent blank (ultra-pure water instead of a urine sample) is processed and analysed as part of each analytical run.

To test for precision, each analytical run includes at least two quality-control samples with a known concentration of the analytes. Since commercial material is not available, the control material must be prepared in the in-house laboratory by spiking urine with the analytes in the relevant concentration range (see Table 7). In addition, control-standard solution (see Section 4.6) and gradient solution (see Section 4.3) are measured as part of each analytical run and rinsing steps with methanol are carried out.

**Tab.7:** Pipetting scheme for the preparation of quality-control samples for the determination of DON and DOM‑1 in urine

Quality-control samples	Urine [μl]	Calibration-standard spiking solution [μl]	Analyte concentration [μg/l]	^13^C_15_‑DON spiking solution [μl]	^13^C_15_‑DON concentration [μg/l]
QC_0.78_	ad 2500	13	0.78	25	1.5
QC_2.4_		40	2.4	25	
QC_5.6_		93	5.6	25	
QC_13.8_		230	13.8	25	
QC_16.8_		280	16.8	25	

## 11 Evaluation of the method

The reliability of this method was confirmed by comprehensive validation as well as by replication and verification of the method in a second, independent laboratory. To determine the precision and accuracy of the method, the quality-control samples (see Table 7) were used, which were processed and analysed according to Section 5 and 6.

To determine the precision and accuracy of the method for DOM‑1, only the quality-control samples QC_0.78_, QC_2.4_, and QC_5.6_ were included and analysed.

### 11.1 Precision

To determine within-day precision, the prepared quality-control materials were processed and analysed six times in parallel at each spiking level. The precision data thus obtained are presented in Table 8.

**Tab.8:** Within-day precision for the determination of DON and DOM‑1 in urine

Analyte	Spiked concentration [μg/l]	Number n	Measured concentration [μg/l]	Standard deviation (rel.) ***s_w_*** [%]	Prognostic range ***u*** [%]
DON	0.78	6	0.81	4.5	11.5
	2.4	6	2.6	2.9	7.5
	5.6	6	5.5	1.5	3.9
	13.8	6	15.7	1.4	3.7
	16.8	6	17.1	1.1	2.9
DOM‑1	0.78	6	0.8	3.8	9.8
	2.4	6	2.4	5.0	12.9
	5.6	6	5.4	4.6	11.7

To determine day-to-day precision, the prepared quality-control materials for DON were processed and analysed on six to eight different days. For DOM‑1, the quality-control samples were processed and analysed on six to seven days. The precision data thus obtained are presented in Table 9.

**Tab.9:** Day-to-day precision for the determination of DON and DOM‑1 in urine

Analyte	Spiked concentration [μg/l]	Number n	Measured concentration [μg/l]	Standard deviation (rel.) ***s_w_*** [%]	Prognostic range ***u*** [%]
DON	0.78	8	0.73	8.5	20.1
	2.4	8	2.5	7.4	17.4
	5.6	6	5.3	3.9	10.0
	13.8	7	14.3	7.9	19.2
	16.8	7	16.7	2.1	5.0
DOM‑1	0.78	7	0.81	3.2	7.9
	2.4	7	2.5	5.1	12.5
	5.6	6	5.4	5.6	14.3

### 11.2 Accuracy

The accuracy of the method was determined from the within-day and day-to-day precision data (cf. Section 11.1). The mean relative recoveries calculated for DON and DOM‑1 are given in Table 10.

**Tab.10:** Mean relative recovery for the determination of DON and DOM‑1 in urine

Analyte	Spiked concentration [μg/l]	Within-day precision			Day-to-day precision		
		Number n	Recovery (rel.) ***r*** [%]	Range [%]	Number n	Recovery (rel.) ***r*** [%]	Range [%]
DON	0.78	6	103	98.0–110	8	93.3	85.7–105
	2.4	6	111	106–114	8	102	93.5–114
	5.6	6	98.8	96.2–100	6	94.9	89.1–98.8
	13.8	6	114	112–115	7	104	89.7–114
	16.8	6	102	100–103	7	99.5	95.6–102
DOM‑1	0.78	6	102	98.6–108	7	103	99.0–110
	2.4	6	101	92.1–108	7	103	94.7–110
	5.6	6	96.9	89.4–102	6	96.6	91.6–104

### 11.3 Limits of detection and quantitation

The detection and quantitation limits given in Table 11 were determined using the calibration-curve method according to DIN 32645; this calculation was based on the six lowest calibration points for DON, whereas all eight calibration points were used for DOM‑1 (DIN 2008). Due to the complexity of the urine matrix, which may be subject to considerable variations, quantitation limits were increased. 

**Tab.11:** Limits of detection and quantitation for the determination of DON and DOM‑1 in urine

Analyte	Limit of detection [μg/l]	Limit of quantitation [μg/l]
DON	0.049	0.179
DOM‑1	0.070	0.260

### 11.4 Analyte stability in the urine matrix

Analyte stability in the urine matrix was investigated at room temperature, at 4 °C, and at −20 °C. Stability at room temperature, which is relevant for sample preparation, was examined for DON and DOM‑1 over a period of 24 hours. Stability during refrigerated storage at 4 °C, which is of interest for short-term storage of urine samples, was examined over a period of 48 hours. Stability at −20 °C is important for longer sample storage and was determined after one week, two weeks, five weeks and 13 weeks.

To determine analyte stability, the quality-control samples were processed and analysed once. Acceptance criteria were based on the Decision 2002/657/EC of the European Union, which allows for a deviation from the nominal value of −50 to +20% (European Commission 2002). The determined relative recoveries of DON and DOM‑1 in urine after storage at room temperature as well as at 4 °C were within the acceptance range (Table 12).

**Tab.12:** Analyte stability of DON and DOM‑1 in urine at room temperature and at 4 °C

Analyte	Spiked concentration [μg/l]	Relative recovery [%] after storage at	
		room temperature for 24 h	4 °C for 48 h
DON	0.78	86.7	87.5
	2.4	99.2	92.3
	13.8	99.9	96.4
	16.8	103	97.5
DOM‑1	0.78	89.1	113
	2.4	91.1	110

The relative recovery of the analytes after storage at −20 °C is presented in Table 13. With the exception of the recoveries of DOM‑1 after one-week storage, all recoveries were found to be within the acceptance range.

**Tab.13:** Analyte stability of DON and DOM‑1 in urine at −20 °C

Analyte	Spiked concentration [μg/l]	Relative recovery [%] after storage at −20 °C for			
		1 week	2 weeks	5 weeks	13 weeks
DON	0.78	86.5	79.4	77.2	91.1
	2.4	94.1	89.6	86.0	87.7
	13.8	96.8	92.9	89.6	85.9
	16.8	100	93.8	89.8	89.5
DOM‑1	0.78	121	113	109	108
	2.4	130	104	115	91.3

### 11.5 Analyte stability in the extract

Analyte stability in the extract of the processed quality-control samples was examined after storage at −20 °C for one week as well as for two, five and 13 weeks. Acceptance criteria were based on the Decision 2002/657/EC of the European Union, which allows for a deviation from the nominal value of −50 to +20% (European Commission 2002). The results of analyte recovery in the extract after storage at −20 °C are presented in Table 14. The recovery for DOM‑1 after the first week of storage as well as the recovery for the quality-control sample QC_0.78_ after two weeks of storage were not within the acceptance range. Moreover, the recovery for DON in the quality-control sample QC_0.78_ after 13 weeks of storage was not within the acceptance range.

**Tab.14:** Analyte stability of DON and DOM‑1 in the extract at −20 °C

Analyte	Spiked concentration [μg/l]	Relative recovery [%] after storage at −20 °C for			
		1 week	2 weeks	5 weeks	13 weeks
DON	0.78	91.7	88.2	90.0	123
	2.4	99.3	101	103	115
	13.8	102	106	103	113
	16.8	104	103	102	112
DOM‑1	0.78	129	127	112	89.8
	2.4	122	113	106	85.4

### 11.6 Sources of error

The method enables the analysis of the total DON content (free DON plus glucuronides), after hydrolysis of the glucu­ronides, as well as the concentration of DOM‑1 in urine. Due to the rapid renal excretion of the substances, the method is primarily suitable for the assessment of an acute exposure just hours before specimen collection.

As part of method development, various SPE materials were tested for purification and enrichment of the analytes. Neither C18 materials nor the polymer phases described in diverse publications could deliver satisfactory results with respect to limits of detection and quantitation.

The detection and quantitation limits described in the literature could be reached by using an immunoaffinity column. The quantitation of DOM‑1 was performed without ISTD, because a linear correlation with the necessary coefficient of determination of R ≥ 0.995 could not be achieved using an isotope-labelled standard in the concentration range investigated.

To prepare the calibration standards, urine from different persons was tested for the levels of the analytes to be measured. As almost all the urine samples analysed contained non-negligible levels of DON, pool urine could not be used. The calibration standards were finally prepared in native urine with almost negligible levels of DON from a person who had eaten a grain-free diet for five days prior to sampling. This urine was collected and stored at –20 °C.

## 12 Discussion of the method

The method enables the reliable determination of DON and its metabolite DOM‑1 in urine. The validation data demonstrate good sensitivity, reproducibility, and accuracy of the method, all of which are the result of efficient sample preparation using an immunoaffinity column. The stationary phase, a gel made of dextranes or cross-linked agarose, was coupled with a suitable ligand (antibody) which specifically binds to the analytes. The antibodies used in the immunoaffinity column exhibited a high specificity for DON. The chromatographic results were excellent; there were no interfering secondary signals and very high recoveries were achieved. The analysis of DOM‑1 described by the developers of the method could not be replicated by the verifiers of the method, as the immunoaffinity column used (IAC DONStarR, Romer Labs Division Holding GmbH, Austria) was discontinued by the manufacturer in April 2021 and replaced by another column (SH-DonStar IAC, specificity 2500 ng DON, No. 10001974, Romer Labs Division Holding GmbH, Austria) that is specific only for DON. The method exhibits high sensitivity and, for DON, is linear across a wide range (up to 21 μg/l urine), such that the method is suitable for application in both environmental and occupational medicine.

At the start of validation, the working ranges were established as 0.3–3 μg/l for both analytes. As a result, the quality-control samples examined for each analyte had concentrations of 0.78 μg/l and 2.4 μg/l urine as well as an ISTD concentration of 1.5 μg/l urine. During validation, the working ranges were expanded and adjusted to real samples from a research project (DON: 0.3–21 μg/l urine, DOM‑1: 0.3–6 μg/l urine). As a result, the quality-control samples additionally examined for DOM‑1 had a concentration of 5.6 μg/l urine; for DON, these concentrations were 5.6 μg/l, 13.8 μg/l, and 16.8 μg/l urine. The concentration of the ISTD was not changed.

**Instruments used** HPLC system with a binary pump, autosampler, column oven, and degasser (Nexera XR, Shimadzu Deutschland GmbH, Duisburg, Germany); triple-quadrupole mass spectrometer (Model AB SCIEX QTRAP 5500 with electrospray ionisation, AB SCIEX Germany GmbH, Darmstadt, Germany)
